# Adjunctive vitamin D therapy in various diseases in children: a scenario according to standard guideline

**DOI:** 10.1186/s12887-022-03297-z

**Published:** 2022-05-07

**Authors:** Hafsa Arshad, Faiz Ullah Khan, Naveed Ahmed, Naveed Anwer, Ali Hassan Gillani, Asim.ur. Rehman

**Affiliations:** 1 Department of Pharmacy Quaid-i-Azam University, Islamabad, 45320 Pakistan; 2 Department of Pharmacy Administration and Clinical Pharmacy, School of Pharmacy, Xi’an Jiaotong University, Xi’an, 710061 China

**Keywords:** Adjunctive vitamin D therapy, Guideline adherence, Socioeconomic effect, Vitamin D supplementation, Comorbidities

## Abstract

**Background:**

Adherence to standard guidelines is imperative when question comes to disease management. The present study aimed to evaluate the administration of adjunctive vitamin D therapy in various diseases, its adherence to standard guideline and the effect of socioeconomic status on the consumption of vitamin D in children.

**Methods:**

Cross sectional observational study was conducted among 400 ambulatory pediatric patients at Children’s Hospital, Pakistan Institute of Medical Sciences Islamabad, from November 2017 to June 2018. Data were collected by a self-designed structured questionnaire from the patient’s medical chart. Adjunctive vitamin D therapy adherence was evaluated by the U. S endocrinology clinical practice guideline of vitamin D deficiency. The association between socioeconomic status and consumption of vitamin D was examined by chi-square. Alpha value (*p* ≤ 0.005) was considered statistically significant. Statistical analysis was done by SPSS version 25.

**Results:**

In 400 patients, 9 diseases and 21 comorbid conditions were identified, in which adjunctive vitamin D therapy was prescribed. Adherence to vitamin D testing in high-risk vitamin D deficiency diseases as; seizures (3.8%), bone deformities (13.3%), steroid-resistant nephrotic syndrome (0.0%), cerebral palsy (5.9%) and meningitis (14.3%). Adherence to prescribed vitamin D dose was in (41.3%) patients in various diseases. Significant association (*p <* 0.05) was found between socioeconomic status and consumption of vitamin D in children and mothers.

**Conclusions:**

It was found that adjunctive vitamin D was being prescribed in various diseases and comorbidities. Overall poor adherence to the standard guideline was observed in disease management in children. Low socioeconomic status affects vitamin D supplementation consumption in children.

**Supplementary Information:**

The online version contains supplementary material available at 10.1186/s12887-022-03297-z.

## Background

The body fulfills vitamin D requirements from a variety of dietary sources and the penetration of UV light in the dermis. Vitamin D deficiency is prevalent in 1 billion people around the globe [1]. Several studies suggested that vitamin D deficiency exacerbate various disease conditions including; osteoporosis, autoimmune diseases, certain cancer, cardiovascular diseases, rickets in pediatrics, osteomalacia, bacterial infections such as tuberculosis, influenza, chronic kidney diseases, and many more [2–5]. Pregnant and nursing women, infants, pediatrics, elder and housebound people are at special risk for developing vitamin D deficiency. A Study conducted in Turkey reported mother vitamin D deficiency associated with infant’s vitamin D level [6]. Social and religious customs, socioeconomic status, illiteracy and skin pigmentation are crucial factors of vitamin D deficiency in the Asian region including Pakistan [7, 8].

Vitamin D deficiency is more prevalent in children, pregnant and nursing women in Pakistan [9], India [10], and Germany [11]. A study reported from Karachi, vitamin D deficiency in 75% of children associated with low socioeconomic communities [12]. Another study conducted in Multan showed severe vitamin D deficiency in 94% nursing mothers [13]. Vitamin D supplementation and vitamin D food fortification products are useful to combat vitamin D deficiency at any age [14]. The UK started the “Healthy Start” program in 2006 to provide free vitamin D supplementation vouchers to low-income pregnant women and children to combat vitamin D deficiency [15]. In Turkey, the “vitamin D prophylaxis augmentation program” started in 2005 to combat vitamin D deficiency in infants. In Pakistan, no such interventional program is available to the population [16]. Different guidelines on vitamin D supplementation are available e.g. Institute of Medicine (IOM) by the USA and Canada [17]; Endocrinology society by USA [18]; Scientific advisory committee on nutrition (SACN) by the UK [19]; European food safety authority (EFSA) [20]; nutritional society of Germany [21]; Polish society of pediatric endocrinology and diabetes [22] clinical practice guidelines by the United Arab Emirates [23]. All these guidelines unanimously recommend vitamin D prescribing according to the patient’s needs and condition of the disease, also emphasize vitamin D consumption during pregnancy and breastfeeding [18, 19, 21–23].

Despite availability of several standard guidelines low adherence to vitamin D prescribing has been seen worldwide even in developed countries. A study conducted in the USA showed poor adherence to vitamin D supplementation in 60% of infants after revised vitamin D guidelines by the American Academy of Pediatrics in 2008 [24]. Another study conducted in Serbia showed poor vitamin D supplementation adherence in osteoporosis in 82% patients [25]. In the UK vitamin testing and prescribing cost is increased 17 folds due to non-adherence to standard guidelines in primary care settings [26]. A study conducted in Turkey reported good adherence to national vitamin D supplementation program to combat vitamin D deficiency in different age groups [27]. Adherence to vitamin D standard guidelines improves the prescribing practices, health outcomes of patients, and disease burden associated with vitamin D deficiency [28]. The unavailability of standard local guidelines in hospital settings is one of the crucial factors for non-evidence-based prescribing [29]. Adherence to standard guidelines is mandatory for adjunctive vitamin D therapy in various disease management in terms of vitamin D testing and prescribed dose. Unfortunately, in Pakistan no such data is available. Most studies have focused on vitamin D deficiency in a particular single disease but to our knowledge, there is a lack of evidence in documenting the adherence to standard guidelines of vitamin D in clinical settings in various diseases along with different co-morbidities especially in Pakistan. To close this gap, this study aimed to evaluate the adjunctive vitamin D therapy in various diseases in children along with different co-morbidities, its adherence to standard guideline and the effect of socioeconomic status on the consumption of vitamin D in children.

## Methods

### Study design and setting

A cross-sectional observational study was conducted at the ambulatory department of the children’s hospital Pakistan Institute of Medical Sciences (PIMS) Islamabad, Pakistan from November 2017 to June 2018. Children’s hospital at (PIMS) is a 230-bed tertiary care hospital providing medical services in various specialties of pediatrics. Approximately 400 patients visit the ambulatory department daily. It is the country’s premier public hospital fulfilling people’s health needs from all over the country.

### Inclusion-exclusion criteria of study population

**Fig. 1:**
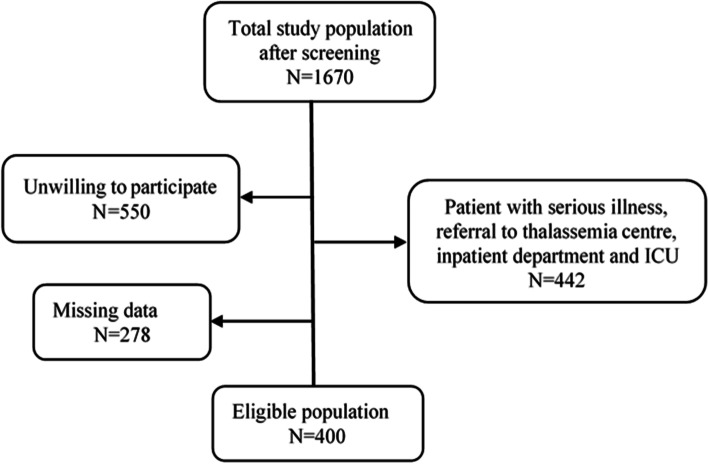
Flow chart of inclusion and exclusion of participants

On initial screening 1670 subjects prescribed with vitamin D, aged ≥3 months to 10 years were recruited from November 2017 to June 2018, approached to the ambulatory department were included in the study. Participants with missing data in their medical records (278), unwilling to participate in study (550), and patient with serious disease conditions e.g. thalassemia, celiac disease, irritable bowel syndrome, leukaemia (to exclude symptoms of diarrhoea and general weakness) and who were referred to thalassemia centre, inpatient department and ICU were excluded (approximately 442) from the study. Finally, 400 patients having vitamin D in their prescription as per pre-defined study protocols were included in the study (Fig. 1).

### Data collection tool

Data were collected through a self-designed structured questionnaire (Additional file 1). The data collection tool was developed after extensive literature review and through expert opinions (supervisory committee of the Department of Pharmacy, QAU, Islamabad). Desired information was extracted from the patient’s medical records. Also, guardians were interviewed to collect information about the patient’s and mother’s vitamin D nutritional parameters. The included *domains* in the data collection self-designed structured questionnaire (Additional file 1) were as: *Patient’s demographics; Patient’s medical history* (diagnosed disease, comorbidities, current vitamin D supplement intake status); *Patient’s feeding practices* (Exclusively breastfeeding (till age of 6 months), Breastfeeding + weaned onto solids (after age of 6 months), Weaned onto solids + cow milk, Weaned onto solids + no milk); *Patient’s vitamin D nutritional parameters* (food fortification product intake, consumption of vitamin D rich diet); *Maternal vitamin D nutritional history* (vitamin D supplement consumption in pregnancy, vitamin D rich diet consumption in pregnancy, vitamin D supplements during breastfeeding); *Clinical laboratory investigations* (vitamin D, serum calcium, serum phosphate, serum alkaline phosphatase, CBC, renal function tests); and *prescribed medications*. In Pakistan no proper guideline is being followed so, adherence to the guideline was evaluated by the U. S endocrinology clinical practice guideline for vitamin D deficiency [18].

### Recommendations of guideline

The U. S endocrinology clinical practice guideline for vitamin D deficiency emphasizes 25- hydroxy vitamin D testing in high-risk diseases in which chances of vitamin D deficiency are higher. In low-risk vitamin D deficiency diseases, vitamin D can be prescribed empirically (400–800 IU) without testing vitamin D level with the advice of lifestyle modification. In high-risk diseases, definitive diagnosis is required after testing vitamin D level, also the dose should be according to confirmed vitamin D level in the body. The 25-hydroxy vitamin D test is a biological indicator to test vitamin D deficiency. Other supportive investigations such as serum calcium, serum phosphate, serum alkaline phosphatase, CBC, and renal function tests are necessary to confirm vitamin D deficiency and to exclude (hypocalcemia and hypercalcemia, hypophosphatemia, hepatic failure, anaemia, renal failure respectively). The guideline also emphasizes on consumption of vitamin D supplementation during pregnancy and breastfeeding (400-800 IU/day). Children are also advised to consume vitamin D food fortification products if not taking vitamin D supplementation properly.

### Guideline adherence evaluation and data analysis

**Fig. 2:**
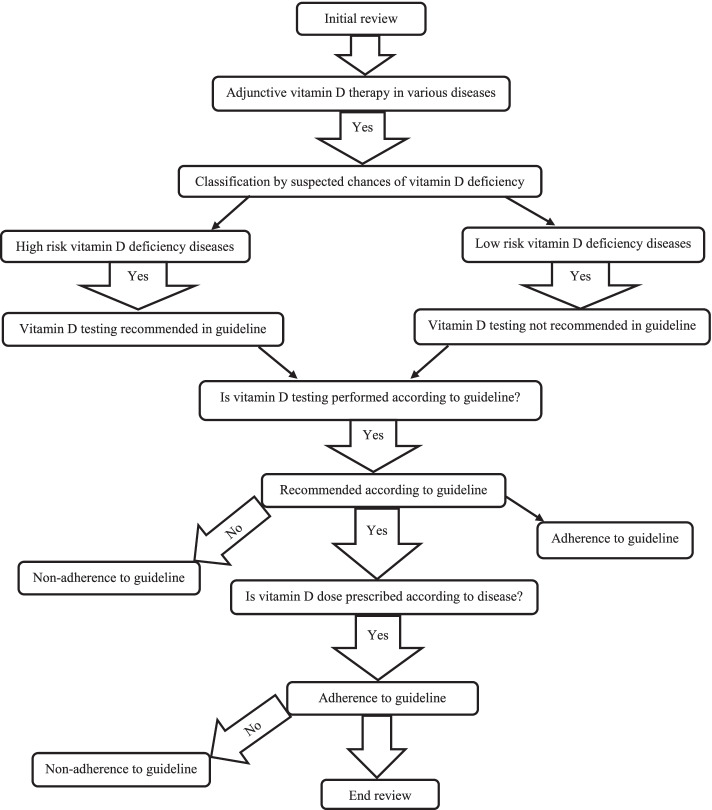
Data evaluation steps to access adjunctive vitamin D therapy (AVDT) adherence to guideline

The initial review of prescriptions was done by a principal investigator, in which vitamin was prescribed in various disease conditions. The diagnosed disease record was extracted from the patient’s medical record through a self-designed structured questionnaire (Additional file 1). Depending upon suspected chances of vitamin D deficiency, diseases were classified as low risk (Respiratory tract infections, urinary tract infections, diarrhea, and general weakness) and high risk (seizures, bone deformities, steroid-resistant nephrotic syndrome, cerebral palsy, meningitis) vitamin D deficiency diseases. The guideline does not recommend 25-hydroxy vitamin D level testing in low-risk vitamin D deficiency diseases. In high-risk vitamin D deficiency diseases, 25-hydroxy vitamin D level testing is recommended. Adherence to vitamin D testing in various diseases along different comorbidities was assessed. Evidence-based dose recommendation was also evaluated (Fig. 2). The dose of vitamin D was evaluated according to the patient’s need by considering the level of vitamin D in the body.

Data were coded and entered through SPSS (IBM version 25). Descriptive statistics including percentages and frequencies for each variable were done. Chi-squared analysis was used to examine the association of socioeconomic status between the consumption of vitamin *D. alpha* value (*p <* 0.05) was considered statistically significant.

## Results

### Patient’s demographics and vitamin D nutritional parameters

Among 400 patients 236 (59.0%) were males. The most patients belonged to age group of (≥ 25 months) 304 (76.0%). Total 211 (52.8%) Patients belonged to the middle class having a monthly income (> 15,000–30,000 PKR). The most common feeding practice in patients was weaned onto solids + cow milk 196 (49.0%). Vitamin D food fortification consumption was only in 44 (11.0%) patients. Currently, 148 (37.0%) patients were taking vitamin D supplements in comorbid conditions. The rest of the other parameters are listed in Table 1.

**Table 1:** Demographics, patient’s medical and nutritional history

Demographics	N (%)	Vitamin D Nutritional Parameters	N (%)
Sex		**Feeding Practices**	
Male	236 (59.0)	Exclusively breastfeeding (till age of 6 months)	27 (6.8)
Female	164 (41.0)	Breastfeeding + weaned onto solids (after age of 6 months)	35 (8.8)
Age Groups (Months)		Weaned onto solids + cow milk	196 (49)
0–12	52 (13.0)	Weaned onto solids + no milk	142 (35.5)
13–24	44 (44.0)	**Vitamin D Food Fortification Consumption**	
		Yes	44 (11.0)
Above 25	304 (70.0)	No	356 (89.0)
		**Consumption of Vitamin D Rich Diet**	
BMI Percentile (WHO, CDC Growth Standards)		Yes	171 (42.8)
< 5 Percentile (underweight)	85 (21.3)	No	229 (57.3)
Percentile ≥5 and < 85 Percentile (Normal weight)	269 (67.3)	**Currently Taking Vitamin D Supplements**	
≥85 and < 95 Percentile (Over weight)	32 (8.0)	Yes	148 (37.0)
≥95 Percentile (Obese)	14 (3.5)	No	252 (63.0)
Economic Status		**Sun Exposure of Child**	
Poor (monthly income < 15,000)^a^	158 (39.5)	Yes	269 (67.3)
Middle Class (> 15,000–30,000)^a^	211 (52.8)	No	131 (32.8)
Rich (> 30,000)^a^	31 (7.8)	**Total**	**400 (100.0)**

^a^*PKR* Pakistani rupees

### Adjunctive vitamin D therapy in various diseases, adherence to standard guideline

In 400 patients, a total of nine diseases; respiratory tract infections (35.8%), seizures (33%), general weakness (28%), bone deformities (15%), diarrhea, vomiting, abdominal cramp (7%), steroid-resistant nephrotic syndrome (6.3%), urinary tract infections (3.8), cerebral palsy (4.3%), meningitis (1.8%), were found in which adjunctive vitamin D therapy was prescribed along with treatment regime (Table 2). These diseases were classified based on suspected chances of vitamin D deficiency, as low risk and high-risk vitamin D deficiency diseases. In respiratory tract infections cases, vitamin D level testing adherence was 101 (70.6%), while dose adherence was in 66 (49.3%) patients. In seizures adherence to vitamin D testing was 5 (3.8%), while dose adherence was in 14 (10.6%) patients. Frequencies and percentages of adherence to the standard guideline for the rest of the diseases are summarized in Table 2.

**Table 2:** Adjunctive vitamin D therapy in various diseases, adherence to standard guideline

Diagnosed Diseases	Classification (Vitamin D Deficiency)	Total N (%)	Guideline Adherence (Vitamin D Testing)		Adherence to Dose (According to Disease)	
			Yes N (%)	No N (%)	Yes N (%)	No N (%)
Respiratory tract infections	Low risk	143 (35.8)	101 (70.6)	42 (29.4)	66 (49.3)	77 (57.5)
Seizure	High risk	132 (33.0)	5 (3.8)	127 (96.2)	14 (10.6)	118 (89.4)
General weakness	Low risk	112 (28.0)	96 (85.7)	16 (14.3)	74 (66.07)	38 (33.9)
Bone deformities	High risk	60 (15.0)	8 (13.3)	52 (86.7)	19 (31.7)	41 (68.3)
Diarrhea, vomiting, abdominal cramp	Low risk	28 (7.0)	24 (85.7)	4 (14.3)	18 (64.3)	10 (35.7)
Steroid resistant nephrotic syndrome	High risk	25 (6.3)	0 (0.0)	25 (100)	6 (24.0)	19 (76.0)
Urinary tract infection	Low risk	15 (3.8)	12 (80.0)	3 (20.0)	13 (86.7)	2 (13.3)
Cerebral palsy	High risk	17 (4.3)	1 (5.9)	16 (94.1)	8 (47.05)	9 (52.9)
Meningitis	High risk	7 (1.8)	1 (14.3)	6 (85.7)	0 (0.0)	7 (100)

### Adjunctive vitamin D therapy in comorbidities, adherence to guideline

Patients having more than one disease were 139 (34.7%) in which adjunctive vitamin D was prescribed in different comorbid conditions. Overall, 21 comorbid conditions were identified in which adjunctive vitamin D therapy was added to the treatment regime (Table 3). Comorbid conditions were classified as low-risk and high-risk vitamin D deficiency comorbid conditions. Most prevalent comorbid conditions were RTIs + general weakness 34 (24.5%), seizures + RTIs 26 (18.7%), RTIs + bone deformities 11 (7.9%), and RTIs +diarrhea 7 (5.0%), in which adjunctive vitamin D therapy was prescribed along with definitive treatment. In RTIs + General weakness, vitamin D testing adherence and dose adherence were in 34 (100.0%) and 21 (61.8%) patients respectively. In seizures + RTIs, vitamin D testing and dose adherence were in 2 (7.7%) and 1 (3.8%) patients respectively. Similarly, in RTIs + Bone deformities vitamin D testing and dose adherence were in 3 (27.2%) and 8 (72.7%) patients respectively. In other comorbid conditions frequencies and percentages, adherence to guideline are listed in Table 3.

**Table 3:** Adjunctive vitamin D therapy in comorbid conditions

Comorbidities	Classification (Vitamin D Deficiency)	Total N (%)	Guideline Adherence (Vitamin D Testing)		Adherence to Dose (According to Disease)	
			Yes N (%)	No N (%)	Yes N (%)	No N (%)
RTIs + General weakness	Low risk	34 (24.5)	34 (100.0)	0 (0.0)	21 (61.8)	13 (38.2)
Seizures +RTIs	High risk	26 (18.7)	2 (7.7)	24 (92.3)	1 (3.8)	25 (96.1)
RTIs + Bone deformities	High risk	11 (7.9)	3 (27.2)	8 (72.7)	3 (27.2)	8 (72.7)
RTIs + Diarrhea	Low risk	7 (5.0)	7 (100.0)	0 (0.0)	4 (57.1)	3 (42.9)
Meningitis + Seizures	High risk	6 (4.3)	1 (16.7)	5 (83.3)	0 (0.0)	6 (100.0)
Steroid resistant nephrotic syndrome + RTIs	High risk	5 (3.6)	0 (0.0)	5 (100.0)	0 (0.0)	5 (100.0)
Diarrhea + General weakness	Low risk	8 (5.8)	8 (100.0)	0 (0.0)	8 (100.0)	0 (0.0)
Urinary tract infection + General weakness	Low risk	5 (3.6)	2 (40.0)	3 (60.0)	3 (60.0)	2 (40.0)
Bone deformities + General weakness	High risk	5 (3.6)	1 (20.0)	4 (80.0)	2 (40.0)	3 (60.0)
Seizures + Bone deformities	High risk	5 (3.6)	0 (0.0)	5 (100.0)	0 (0.0)	5 (100.0)
Cerebral Palsy + RTIs	High risk	4 (2.9)	0 (0.0)	4 (100.0)	1 (25.0)	3 (75.0)
Seizure + General weakness	High risk	4 (2.9)	0 (0.0)	4 (100.0)	0 (0.0)	4 (100.0)
Seizure + Diarrhea	High risk	4 (2.9)	1 (25.0)	3 (75.0)	2 (50.0)	2 (50.0)
Cerebral palsy + Seizures	High risk	3 (2.2)	0 (0.0)	3 (100.0)	1 (33.3)	2 (66.7)
Urinary tract infection + RTIs	Low risk	3 (2.2)	3 (100.0)	0 (0.0)	3 (100.0)	0 (0.0)
Cerebral Palsy + General weakness	High risk	3 (2.2)	0 (0.0)	3 (100.0)	1 (33.3)	2 (66.7)
Bone deformities + Diarrhea	High risk	2 (1.4)	1 (50.0)	1 (50.0)	0 (0.0)	2 (100.0)
Cerebral Palsy + Bone deformities	High risk	1 (0.71)	0 (0.0)	1 (100.0)	1 (100.0)	0 (0.0)
Steroid resistant nephrotic syndrome + Bone deformities	High risk	1 (0.71)	0 (0.0)	1 (100.0)	1 (100.0)	0 (0.0)
Urinary tract infection + Diarrhea	Low risk	1 (0.71)	1 (100.0)	0 (0.0)	1 (100.0)	0 (0.0.)
Meningitis + RTIs	High risk	1 (0.71)	0 (0.0)	1 (100.0)	0 (0.0)	1 (100.0)

### Investigations to test vitamin D deficiency

**Fig. 3:**
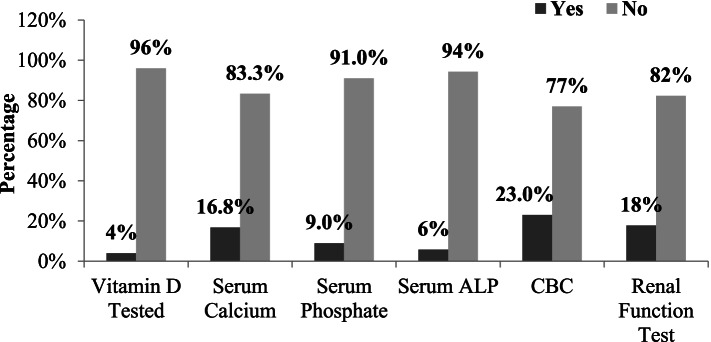
Investigations to test vitamin D deficiency

Percentages of vitamin D deficiency indicator tests, performed in patients were as; vitamin D test, serum calcium, serum phosphate, serum alkaline phosphate, CBC test, renal function test; 16 (4.0%), 67 (16.8%), 36 (9.0%), 23 (5.8), 92 (23.0%), 71 (17.8%) respectively. Percentages of these tests performed and not performed are shown in (Fig. 3).

### Level of 25-Hydroxy vitamin D status

**Fig. 4:**
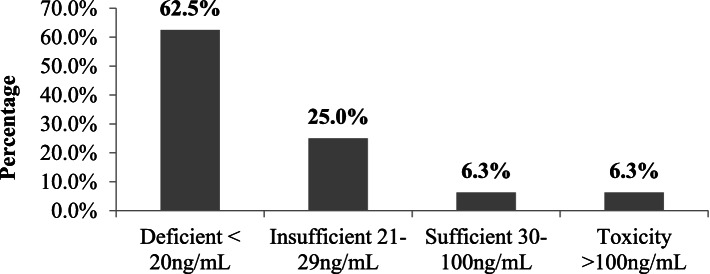
Vitamin D level status

The level of 25-hydroxy vitamin D status was categorized according to the group of endocrinology society in 16 (4.0%) pediatrics patients (Fig. 4) of those patients (62.5%) were vitamin D deficient 20 ng/ml.

### Prescribed vitamin D dose

Adherence to prescribed vitamin D dose was in 165 (41.3%) patients, while in 235 (58.8%) patients there was non-adherence regarding prescribed vitamin D doses in various diseases. 21% of patients were receiving a low dose of vitamin D even less than the dose of empirical therapy (400 IU) (Table 5). 72.5% of patients were receiving a dose of vitamin D between 400 and 1000 IU. In (6.5%) patients the prescribed dose of vitamin D was > 200,000 IU. Other percentages of each dose are listed below in Table 4.

**Table 4:** Prescribed Vitamin D Dose

Prescribed Vitamin D Dose (IU)	Frequency (N)	Percentage (%)
> 200,000	26	6.5
1000	47	11.75
800	11	2.75
700	225	56.25
500	7	1.75
350	84	21

### Maternal vitamin D consumption in pregnancy and breast feeding

**Fig. 5:**
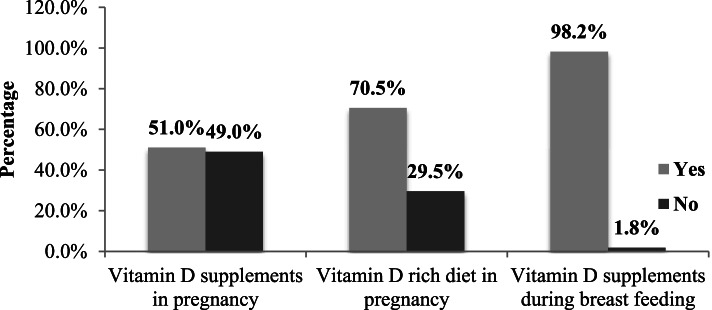
Maternal history about vitamin D supplementation

Mothers who had taken vitamin D supplements and vitamin D rich diet during pregnancy and breastfeeding and who had not taken shown in Fig. 5.

### Reasons for non-consumption of vitamin D supplements and vitamin D rich diet during pregnancy

Mothers who had not taken vitamin D supplements 196 (49.0%) and vitamin D rich diet during pregnancy 118 (29.5%) gave various reasons that are listed in Table 5. In 169 (42.3%) mothers’ duration of breastfeeding was complete 2 years. Sun exposure defined as who exposed to sun light during 10 am to 3 pm and categorized into three main categories 1) 15–30 minutes per day 2) 30–60 minutes per day 3) greater than 1 hour.

**Table 5:** Reasons for non-consumption of vitamin D

Reasons	N (%)	Other Maternal Parameters	N (%)
**Non-consumption of Vitamin D Supplements During Pregnancy**		**Duration of Breast Feeding**	
Not knowing the benefits	92 (46.9)	No feeding at all	74 (18.5)
Cost issue cannot afford	66 (33.7)	Less than 1 year	80 (20.0)
Non-compliance to medication	38 (19.4)	Complete 2 years	184 (46)
Total	196 (100.0)	Currently breastfeeding	62 (15.5)
**No consumption of Vitamin D Rich Diet during Pregnancy**		**Mother’s Sun Exposure During Pregnancy**	
Lack of knowledge about vitamin D diet source	60 (50.8)	Yes	286 (71.5)
Economic issues	43 (36.4)	No	114 (28.5)
Pregnancy complications	15 (12.7)	**Use of sunscreen**	
		Yes	10 (2.5)
**Total**	**118 (100)**	No	390 (97.5)
		Total	400 (100)

### Association between economic status and vitamin D consumption parameters

There was a significant difference between economic status and consumption of vitamin D supplements intake during pregnancy (*p* ≤ 0.003). A significant difference was found between economic status and different vitamin D consumption parameters in mother and children (p ≤ 0.005) listed in Table 6. The percentage of mother’s vitamin D supplementation consumption in pregnancy and breast feeding was 61.3 and 9.7% respectively in high socioeconomic class while 40.5 and 0.6% respectively in poor socioeconomic class. Food fortification products intake in children was 61.3 and 45.6% in high socioeconomic and poor socioeconomic class respectively. Other parameters percentages are listed below in Table 6.

**Table 6:** Association between economic status and vitamin D consumption parameters

Variables	Rich		Economic status Middle		Poor		***P***-value
Vitamin D consumption parameters	Yes N (%)	No N (%)	Yes N (%)	No N (%)	Yes N (%)	No N (%)	*P*-value 95 (%) Cl
Mother’s vitamin D supplements during pregnancy	19 (61.3)	12 (38.7)	121 (57.3)	90 (42.7)	64 (40.5)	94 (59.5)	0.003
Mother’s vitamin D rich diet in pregnancy	25 (80.6)	6 (19.4)	160 (75.8)	51 (24.2)	97 (61.4)	61 (38.6)	0.005
Vitamin D Supplement during breastfeeding	3 (9.7)	28 (90.3)	3 (1.4)	208 (98.6)	1 (0.6)	157 (99.4)	0.002
Child’s food fortification product intake	19 (61.3)	12 (38.7)	9 (4.3)	202 (95.7)	16 (10.1)	142 (89.9)	0.001
Child’s vitamin D rich diet intake	22 (71)	9 (29)	77 (36.5)	134 (63.5)	72 (45.6)	86 (54.4)	0.001

## Discussion

The present study demonstrated the adherence of adjunctive vitamin D therapy in various diseases in children which is the first of its kind in Pakistan. It encompasses all important aspects regarding adjunctive vitamin D therapy in various diseases along with different co-morbidities and socioeconomic effects on vitamin D consumption in children. It will help to understand that proper evidence-based prescribing will help to develop good clinical practices in the future. Also, the study will be helpful to indicate current prescribing practices of adjunctive vitamin D therapy in various disease management along with different co-morbidities in children. Total 9 diseases and 21 comorbid conditions (Tables 2 and 3) were found in which adjunctive vitamin D therapy was prescribed and adherence to the guideline was assessed. Results of this study indicated poor adherence to guideline for adjunctive vitamin D therapy in term of vitamin D testing and prescribed dose in high-risk diseases seizures (5%), steroid-resistant nephrotic syndrome (0.0%), meningitis (14.3%), cerebral palsy (5.9%) and bone deformities (13.3%). The level of 25-hydroxyvitamin D must be tested in high-risk vitamin D deficiency diseases according to recommended guidelines [18, 23]. Only 4% of patients were tested for their 25-(OH) D level in all diseases indicating poor adherence to the guideline. Results of this are in line with a study conducted in Sweden indicating poor guideline adherence with Vitamin D level testing in high-risk diseases [30]. A study conducted by Uday S et al., across 29 countries, infant vitamin D supplements policies adherence rank was better in Austria and Hungary (98%), Netherland, France and Estonia (90%), Russia (80%), Germany (90–70%), Norway (70%) while Denmark (60–70%), Ireland (59%), Greece (30%), and UK (20%) has low rank to vitamin D supplementation policies adherence at national level [31].

Empirically vitamin D was added to therapy in low-risk vitamin D deficiency diseases e.g. RTIs (70.6%), general weakness (85.7%), urinary tract infections (80%), diarrhea (64.3%) without testing 25-hydroxyvitamin D levels because it is an expensive test. A Survey conducted in India reported vitamin D was prophylactically prescribed in infants according to the Indian academy of pediatrics (25.6%) and the American academy of pediatrics (19.2%). These results are contradicting with the present study in which slightly better adherence was seen in low risk diseases [32].

Twenty-one (21) comorbidities (Table 3) were identified as low-risk and high-risk comorbidities in which vitamin D was adjunctively prescribed. In comorbid conditions, chances of vitamin D deficiency are higher due to the cumulative effect of individual disease. According to recommendations in comorbid conditions confirmed vitamin D testing is necessary for definitive diagnosis [18]. Since vitamin D deficiency cannot be compensated with an empirical maintenance dose of 400–1000 IU. Overall poor compliance to guidelines was seen in this study. A population-based cohort study conducted in the UK from The Health Improvement Network (THIN) database records shows similar results that vitamin D prescribing in children has increased 26 folds from 2008 to 2016 without consistent guideline’s recommendations in terms of the prescribed dose and inappropriate testing [33]. The main reasons for poor guideline adherence to vitamin D testing were due to unavailability of national guideline in government hospitals and physicians’ knowledge gap about standard vitamin D guidelines. In developing countries including Pakistan unavailability of proper laboratory facilities in government hospitals and the unavailability of vitamin D supplements are the main reasons for compromised treatment [32].

Vitamin D supplementation intake is recommended in pregnant and nursing women. In our study, most patients belonged to low socioeconomic class. The nutritional status of children and their mothers was compromised due to their low-income status. In this study, a significant association was found between economic status and mother’s vitamin D supplements during pregnancy (*p* = 0.003), vitamin D supplement during breastfeeding (*p* = 0.002) mother’s vitamin D rich diet in pregnancy (*p* = 0.005). Vitamin D supplements intake and vitamin D-rich diet during pregnancy and breast-feeding were affected due to low socioeconomic status, because most mothers did not know about the benefits of vitamin D supplementations during pregnancy. Vitamin D supplements are a little bit expensive and this socioeconomic class could not afford the vitamin D supplements. Low education level and cost were the core issues. A study conducted in India, showing similar results of the association between vitamin D supplements intake during pregnancy and low socioeconomic status in pregnant women (*p <* 0.001) [34]. In a lower socioeconomic class, the trend of breastfeeding was higher in this study. Most mothers (46%) breastfed their child for the complete 2 years. Meanwhile, only 2% of mothers had taken vitamin D supplementation during breastfeeding so, suspected chances of vitamin D deficiency were higher in these children because human milk has low vitamin D content [35]. According to the recommendation by standard guidelines pregnant and breastfeeding mothers should take 400 IU of vitamin D supplements in these conditions [18, 19, 21–23].

In this study, low economic status has a significant association with consumption of vitamin D food fortification products and vitamin D rich diet (egg yolk, fish, liver, cheese, and beef) in children in this study (0.001). Food fortification products are expensive and low economic status affects its consumption in Pakistan as well as in developing countries as compared to developed countries. A study conducted in Saudi Arabia (children 378, age 2–20 years) contradicted with our results in which low socioeconomic status was not significantly associated with consumption of vitamin D rich diet and vitamin D fortified food [36]. A cross-sectional survey conducted in the USA by from National Health and Nutrition Examination Survey (NHANES) database on different ethnic groups (8214 children, age less than 19 years) showed vitamin D dietary intake was 3 times higher in high-income Hispanic families than low-income Hispanic group and non Hispanic blacks, results of this study are aligned with our study [37]. A meta-analysis of 20 randomized control trials conducted by Khalifah et al., suggests vitamin D food fortification effectively improves vitamin D status in children [38].

## Conclusion

Overall low adherence to the standard guideline was found in terms of vitamin D level diagnosis and prescribing. Although, adjunctive vitamin D therapy was being prescribed in disease management but there was a lack of evidence-based prescribing. In comorbidities, vitamin D level testing and prescribed dose were not in compliance with guidelines. The patient’s nutrition status was compromised due to low socioeconomic status, low education status, and less awareness about the importance of vitamin D. Due to low socioeconomic status and lack of mother’s knowledge about vitamin D rich diet, mothers had not taken vitamin D rich diet and vitamin D supplementation during pregnancy and breastfeeding. It is recommended that evidence-based prescribing according to standard guidelines protocols should be adopted by health care professionals. Free approachable vitamin D testing facilities must be provided to the population at the government level to cope with increasing vitamin D deficiency. Provision of local guidelines in hospitals, interventional programs, and training must be conducted at regular intervals for health care professionals to emphasize the implementation of standard guidelines.

This study evaluated the diverse aspects of vitamin D as adjunctive therapy in various disease conditions in children concern to standard guideline. The novelty of this study was well demonstrated by the various aspects of vitamin D in disease management, comorbidities, feeding practices of children, mother’s consumption of vitamin D in pregnancy and breastfeeding, this study tried to cover every important aspect of vitamin D consumption as adjunctive therapy to treat various diseases. A diverse study population minimized predictability bias. Moreover, due to the small sample size and limited time frame, findings cannot be generalized to the whole population. Disease association to vitamin D level cannot be determined because laboratory data was insufficient to find any conclusion. Due to a cross-sectional study causal effect cannot be determined.

## Supplementary Information

**Additional file 1.** Self-Designed Structured Questionnaire

## Data Availability

The datasets used and/or analyzed during the current study are available from the corresponding author on reasonable request.

## Abbreviations

IOM: Institute of Medicine
SACN: Scientific Advisory Committee on Nutrition
CBC: Complete Blood Count
AVDT: Adjunctive Vitamin D Therapy
SD: Standard Deviation
RTIs: Respiratory Tract Infections
Cl: Confidence Interval
IU: International Units
