# Supplementary material for: Adjunctive vitamin D therapy in various diseases in children: a scenario according to standard guideline
Source: BMC Pediatr. 2022 May 7;22:257. doi: 10.1186/s12887-022-03297-z (PMC9077968; doi:10.1186/s12887-022-03297-z)
Supplement: Supplementary file 1 — Additional file 1. Self-Designed Structured Questionnaire [file 12887_2022_3297_MOESM1_ESM.docx]

# **1. Patient information/ Demographics**

| **Ward/OPD**_______ | **Date:** _______ | **Economic status** | **Contact no.**_______ |
| --- | --- | --- | --- |
| - Male - Female | - Age _________ - Height ________ - Weight________ - BMI Percentile______ | - Poor (monthly income <15000) - Middle Class (>15000-30000) - Rich (>30000) | Address:_____________   - City ____________ - Province ________ |

**2. Child’s Medical History**

| Medical history | Gestation period | **Current diagnosed disease** | **Medication taking before diagnosis** |
| --- | --- | --- | --- |
| **Allergy**   - Yes - No | - Normal - Premature - Delayed | - __________________ - **Comorbidity**_______ | - No - Yes |

**3. Child Nutrition History**

| 1. **Feeding practice of child**  - Exclusively breast feeding - Breast feeding + weaned onto solids - weaned onto solids + cow milk - weaned onto solids + no milk  1. **Is child taking any food fortification product?**  - Yes - No  1. **Regular use of vitamin D rich food e.g. egg, milk, fish and dairy products?**  - Yes - No - Reason _______________ | 1. **Sunlight exposure of child?**  - Yes - No   **If yes, how much time?**   - 15-30 min per day, - 30-60 min per day, - greater than 1 hour  1. **Use of sun screen?**  - Yes - No  1. **Currently taking vitamin D supplements?**  - Yes - No |
| --- | --- |

**4. Maternal History**

| 1. **Did mother take vitamin D Supplements during pregnancy?**  - Yes - No - Reason _____________  1. **Did mother take vitamin D supplements during breast feeding?**  - Yes - No  1. **Duration of breast feeding**  - No breast feeding at all - Less than 2 years - Complete 2 years - Currently breast feeding | 1. **Did mother take Vitamin D rich diet during pregnancy?**  - Yes - No - Reason__________  1. **Sunlight exposure of mother?**  - Yes - No  1. **Use of sun screen?**  - Yes - No |
| --- | --- |

**5. Lab investigations**

| 1. **Serum (25-OH D**)  - Performed - Not performed   **Adherence to guideline**   - Yes - No   **Vitamin D level status**   - Deficient - Insufficiency - Sufficient - Toxicity | 1. **Serum calcium (Ca2+)**  - Performed - Not performed   **Adherence to guideline**   - Yes - No | 1. **Serum phosphate**  - Performed - Not performed   **Adherence to guideline**   - Yes - No |
| --- | --- | --- |
| 1. **Serum ALP**  - Performed - Not performed   **Adherence to guideline**   - Yes - No | 1. **Blood Count**  - Performed - Not performed   **Adherence to guideline**   - Yes - No | 1. **Renal function test**  - Performed - Not performed   **Adherence to guideline**   - Yes - No |

**6. Prescribed medications**

| **No.** | **Drug** | **Does** | **Frequency** | **Vitamin D Dose Adherence to Guideline** |
| --- | --- | --- | --- | --- |
| **1.** |  |  |  |  |
| **2.** |  |  |  |  |
| **3.** |  |  |  |  |
| **4.** |  |  |  |  |
| **5.** |  |  |  |  |
| **6.** |  |  |  |  |

**Any further comments/ observations**

|  |
| --- |
